# Role of brain 2-[^18^F]fluoro-2-deoxy-D-glucose-positron-emission tomography as survival predictor in amyotrophic lateral sclerosis

**DOI:** 10.1007/s00259-022-05987-3

**Published:** 2022-10-29

**Authors:** Antonio Canosa, Alessio Martino, Umberto Manera, Rosario Vasta, Maurizio Grassano, Francesca Palumbo, Sara Cabras, Francesca Di Pede, Vincenzo Arena, Cristina Moglia, Alessandro Giuliani, Andrea Calvo, Adriano Chiò, Marco Pagani

**Affiliations:** 1 ALS Centre, “Rita Levi Montalcini” Department of Neuroscience, University of Turin, Via Cherasco 15, 10126 Turin, Italy; 2 SC Neurologia 1U, Azienda Ospedaliero-Universitaria Città della Salute e della Scienza di Torino, Turin, Italy; 3 Institute of Cognitive Sciences and Technologies, C.N.R., Rome, Italy; 4 Department of Business and Management, LUISS University, Viale Romania 32, 00197 Rome, Italy; 5 Positron Emission Tomography Centre AFFIDEA-IRMET S.p.A., Turin, Italy; 6 Environment and Health Department, Istituto Superiore di Sanità, Rome, Italy; 7 Neuroscience Institute of Turin (NIT), Turin, Italy; 8 Department of Medical Radiation Physics and Nuclear Medicine, Karolinska University Hospital, Stockholm, Sweden

**Keywords:** Amyotrophic lateral sclerosis, 2-[^18^F]FDG-PET, Survival, Prediction model

## Abstract

**Purpose:**

The identification of prognostic tools in amyotrophic lateral sclerosis (ALS) would improve the design of clinical trials, the management of patients, and life planning. We aimed to evaluate the accuracy of brain 2-[^18^F]fluoro-2-deoxy-D-glucose-positron-emission tomography (2-[^18^F]FDG-PET) as an independent predictor of survival in ALS.

**Methods:**

A prospective cohort study enrolled 418 ALS patients, who underwent brain 2-[^18^F]FDG-PET at diagnosis and whose survival time was available. We discretized the survival time in a finite number of classes in a data-driven fashion by employing a *k*-means-like strategy. We identified “hot brain regions” with maximal power in discriminating survival classes, by evaluating the Laplacian scores in a class-aware fashion. We retained the top-*m* features for each class to train the classification systems (i.e., a support vector machine, SVM), using 10% of the ALS cohort as test set.

**Results:**

Data were discretized in three survival profiles: 0–2 years, 2–5 years, and > 5 years. SVM resulted in an error rate < 20% for two out of three classes separately. As for class one, the discriminant clusters included left caudate body and anterior cingulate cortex. The most discriminant regions were bilateral cerebellar pyramid in class two, and right cerebellar dentate nucleus, and left cerebellar nodule in class three.

**Conclusion:**

Brain 2-[^18^F]FDG-PET along with artificial intelligence was able to predict with high accuracy the survival time range in our ALS cohort. Healthcare professionals can benefit from this prognostic tool for planning patients’ management and follow-up. 2-[^18^F]FDG-PET represents a promising biomarker for individual patients’ stratification in clinical trials. The lack of a multicentre external validation of the model warrants further studies to evaluate its generalization capability.

**Supplementary Information:**

The online version contains supplementary material available at 10.1007/s00259-022-05987-3.

## Introduction

Amyotrophic lateral sclerosis (ALS) is a progressive degenerative disorder affecting motor neurons and leading to loss of function in bulbar and spinal muscles. Death usually occurs within 2 to 5 years after the onset, mainly due to respiratory failure [1]. A wide range of demographic and clinical features have been shown to negatively impact ALS prognosis [2], including older age, bulbar onset, shorter time delay from onset to diagnosis, definite El Escorial level [3] at diagnosis, and cognitive-behavioral impairment [4]. The identification of prognostic tools for ALS is a major area of research since they would improve the design of clinical trials and the clinical management of patients. Several prognostic models have been proposed, but most of them show methodological pitfalls, thus limiting their applicability [5]. Some studies [6, 7] based on brain 2-[^18^F]fluoro-2-deoxy-D-glucose-positron-emission tomography (2-[^18^F]FDG-PET) reported that extensive hypometabolism in frontotemporal areas is independently associated with shorter survival. A more recent investigation highlighted that bulbar hypermetabolism at 2-[^18^F]FDG-PET was associated with shortened survival in patients with ALS or motor neuron disease associated with frontotemporal dementia [8]. The prognostic value of magnetic resonance imaging (MRI) in combination with clinical features has also been investigated, supporting the inclusion of neuroimaging in prognostic models [9, 10].

In this study, we aimed to evaluate in a very large dataset of ALS patients the accuracy of brain 2-[^18^F]FDG-PET, performed at the time of diagnosis, as an independent biomarker of survival.

## Methods

### Participants

We included 418 consecutive patients diagnosed with definite, probable, and probable laboratory-supported ALS according to El Escorial revised diagnostic criteria [3] between 2009 and 2019 at the ALS Expert Centre of Turin, Italy, who underwent brain 2-[^18^F]FDG-PET at the time of diagnosis and whose survival time (i.e., time from PET to death/tracheostomy) was available. The following demographic and clinical characteristics were collected: age at PET, sex, site of onset (spinal/bulbar), and King’s stage at PET [11]. King’s stages 4a and 4b were combined as stage 4.

### 2-[^18^F]FDG-PET image acquisition and pre-processing

Brain 2-[^18^F]FDG-PET was performed according to published guidelines [12]. Patients fasted at least 6 h before the exam. Blood glucose was < 7.2 mmol/l in all cases before the procedure. After a 20-min rest in a dim light and silent room, with eyes closed and ears unplugged, about 185 MBq of 2-[^18^F]FDG was injected. The acquisition started 60 min after the injection. PET/CT scans were performed on a Discovery ST-E System (General Electric). Brain CT and PET scan were sequentially acquired, the former being used for attenuation correction of PET data. The PET images were reconstructed with four iterations and 28 subsets with an initial voxel size of 2.34 × 2.34 × 2.00 mm and data were collected in 128 × 128 matrices.

Images were spatially normalized to a customized brain 2-[^18^F]FDG-PET template [13] and subsequently smoothed with a 10-mm filter in Matlab R2018b (MathWorks, Natick, MA, USA). Intensity normalization was performed at the individual level averaging each voxel for the mean value of the whole brain. Normalization with a subcortical reference region was not considered as an option, since all brain regions have been demonstrated to be potentially affected in ALS.

Since further analyses considered each voxel as a single feature, by using automated anatomical labeling atlas (https://www.gin.cnrs.fr/en/tools/aal/) only the whole brain volume (i.e., the cerebrum, the brainstem, and the cerebellum) has been retained from entire PET scans reducing the amount of candidate voxels from 91 × 109 × 91 = 902629 to 226954 voxels.

### Analysis of variance (ANOVA)

In order to assess whether survival time correlated with other clinical and demographic information two different ANOVA tests were performed, considering the survival time as a continuous dependent variable and sex and site of onset as categorical independent grouping variables. Statistical significance was set at *p <* 0.05. Details on the ANOVA test can be found in the Online Resource (Section 1). To take into consideration the deviation from a Gaussian shape of survival time, a log-rank test as applied to Kaplan-Meier plots was implemented. A similar approach, using Cox proportional hazard regression analysis, was performed to assess the relationship between survival time and age at PET.

### Determining the survival time profiles

Survival time is intended as a real-valued continuous variable. To cast the survival time prediction problem as a supervised classification problem (i.e., a pattern recognition with a finite number of categorical classes), it is necessary to discretize the survival time in a finite number of classes. The soundness of the discretization is addressed by evaluating the percentage of variance explained by the *k*-means unsupervised classification technique adopted to generate classes. The *k*-means clustering generates, for each a priori choice of number of classes *k*, the solution maximizing the between-clusters variance. We then selected the solution with *k* (number of classes) in which the ratio between clusters/within-cluster variance (formally identical to an R-square) was maximal. The rationale behind this approach is that we let the survival time groups to automatically emerge in a data-driven fashion from the available cohort instead of relying on any a priori (manual, and possibly biased) discretization. Mathematical details on how to calculate the variance explained (*VE*) of a partition of *n* samples into *k* groups (i.e., survival time profiles) are reported in the Online Resource (Section 2).

### Classification of survival times

The aims of the pattern recognition analysis in this work were: first, to classify, hence predict, the survival time based solely on the brain PET scan at diagnosis; second, to identify “hot regions” within the brain (i.e., clusters of relevant voxels) with maximal power in discriminating survival profiles.

The latter point is crucial for this analysis and perfectly frames the proposed pattern recognition pipeline within the recent explainable artificial intelligence (XAI) umbrella [14]. To this end, the proposed procedure is composed of the following three steps: first, an unsupervised feature selection (i.e., voxel selection) phase; second, assessment of the meaningfulness of the selected features according to the classifier; third, a posteriori validation of the selected features.

As regards the first step, since the search space is huge (226954 candidate features), evolutionary or greedy heuristics are likely to fail to converge to a suitable solution due to high probability of being trapped in local solutions. For this purpose, our choice fell on leveraging filter methods and, in particular, on Laplacian score [15], an unsupervised technique for feature ranking which rewards features that preserve the local connectivity among patterns, provided that a similarity graph can be constructed. To identify significant features for each of the *k*-classes, the Laplacian scores have been evaluated for each group of patients independently. This procedure yields different groups of features for different classes. For further details on Laplacian scores, see the Online Resource (Section 3). Once the Laplacian scores have been evaluated, it is possible to retain the top-*m* features for each class and use them to train the classification systems. To obtain pathophysiologically interpretable results, we considered as significant only clusters including more than 100 contiguous voxels.

As a classification system, a support vector machine (SVM) emerged as the most performing classification system after a comparison with K-nearest neighbors (K-NN), decision tree, random forest, and a linear classifier (comparison data are reported in the Online Resource, Section 4, eTable 1). Since the classification problem has *k* classes (each one is characterized by a different set of features), a one-against-all approach [16] has been employed to train the classifiers: a given class is marked as “positive” and all other classes are marked as “negative.” As common for machine learning models, SVMs need a careful hyper-parameter tuning for the data and the problem under analysis, and Bayesian optimization [17] has been used to perform such hyper-parameter tuning in an automatic fashion. The latter has been configured to run for a maximum number of 3000 iterations to optimize the regularization term, the kernel type, and (eventually) the kernel parameters of the SVMs. The objective function to be maximized is the 10-fold cross-validation informedness (see Online Resource, Section 5) evaluated on a training set composed by 90% of the overall number of subjects. At the end of the optimization phase, each classification system is re-trained with the optimal hyper-parameters and its final performances are evaluated on a separate test set (the remaining 10% of the overall number of subjects).

### Demographic and clinical distribution within the survival profiles

Given the *k* classes of survival, we investigated the distribution of both demographic and clinical information among the *k* groups, considering patients’ mean age at PET, sex ratio (male/female), and site of onset.

### Comparative analysis between survival times and King’s stages

We evaluated whether a correlation between the King’s stages and survival exists. We performed a one-way ANOVA grouping by King’s stage, from one to four. We also evaluated the odds ratio (OR) between being in one of the King’s stages against being in one of the *k* survival time profiles.

## Results

### Clinical characteristics and correlation with survival

Our cohort included 418 patients (177 female, 42.4%), of whom 290 (69.4%) had spinal onset. The median age at PET was 67 years (Interquartile Range, IQR, 58–73), and median survival was 1.45 years (IQR 0.61–3.27).

ANOVA showed a significantly longer survival in females (*F* = 14.67, *p* = 0.0001), confirmed by the Kaplan-Meier plot (log-rank test *p* = 0.0002; eFigure 1, Section 6 in the Online Resource). No difference was found when site of onset was considered. Finally, the Cox proportional hazard regression analysis demonstrated a negative correlation between survival time and age at PET (*p* = 0.0012; regression coefficient estimate, *b* = 0.0147).

### Classification of survival times

Data are discretized by an unsupervised procedure in three clear-cut survival profiles: a first class with survival range (0–2) years, a second class with survival range (2*–*5) years, a third class with survival > 5 years.

As intuitable by visual inspection (see eFigure 2, Section 6 in the Online Resource), this *k*-means-like data-driven discretization of the (continuous) survival times defines a percentage of variance explained (VE) of 92%, hence it can be considered as reliable for determining the *k* = 3 survival classes to solve the supervised classification problem.

Based on the feature ranking strategy of Laplacian scores, a common trend emerged across the three classes: the first 2000 features (i.e., voxels) held the higher scores and an even smaller number (i.e., 400 voxels) of features was still able to preserve the vast majority of information.

The SVMs, subject to Bayesian optimization to maximize the 10-fold cross-validation informedness, resulted in an error rate (evaluated on the test set) of 19.51%, 24.39%, and 4.87% for the three classes separately (i.e., c-statistics of 0.81, 0.78 and 0.98, respectively). The K-NN emerged as the second most performing technique, able to outperform SVMs on class 2 only (error rate 19.51% and c-statistics of 0.74).

**Fig. 1:**
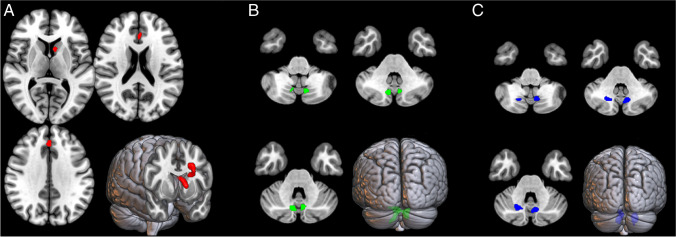
Binary depiction of clusters of discriminating voxels for the three classes. Class one (left, red, **A**), class two (central, green, **B**), and class three (right, blue, **C**). Two-dimensional (2D) representation of transversal slices and 3D rendering projections of the whole brain according to the MNI referential atlas (MRIcroGL software, https://www.nitrc.org/projects/mricrogl). For class one brain rendering was clipped at the level of anterior cingulate cortex and caudate nuclei

Figure 1 and Table 1 show the clusters of relevant voxels for the three classes.

**Table 1:** Clusters of relevant voxels for the three survival classes

Survival class	Cluster extent	Talairach coordinates			Cluster site
		*x*	*y*	*z*	
1	121	−10	10	11	Left caudate body
	166	−2	37	26	Left anterior cingulate, Brodmann area 32
2	466	−4	−65	−23	Left cerebellum, anterior lobe, pyramid
3	180	16	−62	−26	Right cerebellum, anterior lobe, dentate nucleus
	265	−9	−63	−24	Left cerebellum, anterior lobe, nodule

Survival class one, two, and three correspond to 0–2, 2–5, and > 5 years respectively

As for class one, the two discriminant clusters included left caudate body and left anterior cingulate cortex (Fig. 1A). Using the mean whole brain metabolism as a reference, the former cluster showed a relative hypometabolism, while the latter was relatively hypermetabolic. Relative hypermetabolism was also found in the clusters of the other two classes, in which the most discriminant regions were bilateral cerebellar pyramid in class two (Fig. 1B), and right cerebellar dentate nucleus and left cerebellar nodule in class three (Fig. 1C).

Table 2 reports the demographic and clinical descriptive statistics for each of the three classes.

**Table 2:** Demographic and clinical descriptive statistics for the three survival classes

Survival class	Age at PET (years)	Sex	Onset	King’s 1	King’s 2	King’s 3	King’s 4	*n*
1	68 (IQR 74–59)	161 M/94 F	85 B/170 S	88	74	88	11	255
2	65.5 (IQR 71.5–56.5)	66 M/58 F	32 B/92 S	62	37	21	4	124
3	64 (IQR 70–50.25)	14 M/25 F	10 B/29 S	20	14	5	0	39

Abbreviations: *IQR* Interquartile Range, *M* male, *F* female, *B* bulbar, *S* spinal

### Assessment of correlation between survival profiles and King’s stages

One-way ANOVA exploring the correlation between survival profiles and King’s stages resulted in a significant difference (*F* = 4.18, *p* = 0.0062; eFigure 3, Section 6 in the Online Resource). The ORs were consistent with the ANOVA results. In King’s 1 we found a decrease in odds of being part of class one (OR 0.52, CI 0.35–0.78) and a parallel increase of being part of class two (OR 1.72, CI 1.13–2.63). Conversely, King’s stage 3 showed a complementary pattern (OR for class one 2.50, CI 1.52–4.10; OR for class two 0.49, CI 0.28–0.83). King’s 2 did not show any statistically significant ORs.

## Discussion

The main added value of the present study is the evidence of the ability of brain 2-[^18^F]FDG-PET along with artificial intelligence (AI) to independently predict with high accuracy the survival time range in ALS patients. This goal was achieved by investigating the largest ALS series studied so far by PET in which patients’ scans were obtained by the same equipment and acquisition protocol, assuring the homogeneity of the data. This study is reported in agreement with the TRIPOD statement for transparent reporting of prediction models [18].

A recent review [5] about prognostic models for ALS reported 19 studies focused on survival. None of these studies matches our approach. In fact, most of the studies exploited clinical features, in some cases derived from public databases. When available, the c-statistics (i.e., the area under the curve) ranged from 0.74 to 0.87. Among the cited works, only two [19, 20] used an external validation cohort, whereas the others relied on internal validation. Of the two studies [9, 10] in which neuroimaging was used alongside clinical features, only one [10] included c-statistics. This study proposed a clinical and MRI multivariable algorithm, including diagnostic delay and semantic fluency, resulting in an area under the curve of 0.77. Overall, the review [5] concluded that only the ENCALS prognostic model [19] was at low risk of bias and therefore relatively reliable for clinical practice, with an external predictive accuracy of 0.78. The ENCALS model is based on eight clinical and genetic parameters and is available online (http://www.encalssurvivalmodel.org). In addition to the very high predictive capability (about 80%, but up to 95% in the long surviving class), with c-statistics for the proposed SVMs ranging from 0.78 to 0.978, which outperforms competing works, major differences of our model from previous works include: first, lack of feature engineering or any manual choice of the features (the most discriminative clusters for the three survival classes automatically emerged from the pattern recognition pipeline); second, use of features extracted from a single biomarker allowing an independent prediction of the clinical outcome.

The novelty of our study mainly resides in the choice of utilizing neuroimaging only in a way that can be meaningful for clinicians to sketch hypotheses on the prognostic evolution of the disease in relation to the metabolic status at the first visit. This was made possible by the adoption of a high explainability (white-box) pattern recognition approach.

The possibility of predicting patients’ survival should be handled cautiously, considering the potential implications for clinicians, patients, and their families. In this respect, healthcare professionals would benefit from such a tool for planning patients’ management and follow-up. On the other hand, the choice of patients and their relatives to receive a prediction of survival depends on their psychological and personal characteristics, along with their cultural and social background [19]. Patients’ awareness can facilitate life-planning, but unwanted information can hamper their hope and quality of life. Ultimately, patients’ information should meet their personal needs.

In a research setting a potentially important innovation from our model might be the improvement of clinical trial design, allowing patients’ stratification based on a prognostic predictive biomarker. Indeed, the huge clinical heterogeneity of ALS is one of the factors underpinning the disappointing results of most ALS clinical trials of the last three decades [21]. A proposed strategy is to include a measure of the disease progression rate (e.g., ΔALSFRS-R=points lost per month) among selection criteria, but this approach does not assure to include homogeneous populations in clinical trials [22]. The guidelines of the 2019 Revised Airlie House consensus for the design and implementation of ALS clinical trials suggested the use of predictive biomarkers [23]. Indeed, replacing group-level criteria with tailored criteria relying on prognostic modeling strategies can address many of the current trial design limitations [22].

In the field of AI applied to medical imaging, ethical debates have arisen [14]. Crucial issues are the explicability of the results provided by AI methods and the need to reduce their “black-box” character. The latter concept refers to the situation in which the outputs of the algorithm are not understandable. Significantly, our model identified discriminant clusters in brain regions already known to be involved in ALS neurodegeneration. According to a neuropathological staging system [24], the neurodegenerative process spreads via the axonal pathways in a sequential pattern in four neuropathological stages. In this staging system, caudate nucleus is reported to be involved in a relatively advanced phase of the disease, providing an explanation for our observation of its relative hypometabolism in discriminating patients with the worst survival profile. Also, the anterior cingulate cortex is known to be involved in ALS, and pathological data suggest its implication in advanced stages [25]. Cerebellar involvement in ALS has been extensively confirmed by several 2-[^18^F]FDG-PET studies [6, 26]. Cerebellar adaptive changes have been reported in a wide range of neurological conditions [27], providing a possible explanation of the finding of clusters of cerebellar relative hypermetabolism discriminating the two classes with a longer survival.

We found that an older age was associated with worse survival, in keeping with published literature [2], while we did not find any significant correlation with the site of onset, differently from previous studies [2]. Bulbar onset has been extensively demonstrated to be a negative prognostic factor in ALS in population-based cohorts. Otherwise, our sample is not population-based. The lack of a negative effect on survival in our study could be due to the lower-than-expected bulbar/spinal ratio in the lower survival class. A higher attrition rate among bulbar patients with a more severe disease at diagnosis could underlie this result. Additionally, we could not rule out an unbalanced distribution of other not considered prognostic factors among spinal and bulbar patients in the same survival class. Regarding King’s staging, the small size of stages three and four dramatically widens the relative confidence intervals, preventing the identification of any statistical significance. However, no King’s 4 patients are included in the long-surviving class three, while King’s 1 patients span the entire range of survival time classes with similar relative frequency across the three classes. This ends up into an uncertainty (maximal entropy) of survival time prediction starting from a King’s 1 stage, suggesting a non-linear deterioration of ALS. On the other hand, an approach based on (explainable) artificial intelligence reduces the uncertainty of the survival time prediction based on brain PET scan.

Some radiopharmaceuticals other than 2-[^18^F]FDG can provide interesting information about microglial activation or synaptic density, e.g., (*R*)-[^11^C]PK11195 and [^11^C]UCB-J respectively, and might be employed to evaluate their possible role in prognostic prediction. Nevertheless, the fact that they are marked with [^11^C] makes their use not feasible on a wide scale. Therefore, we focused on 2-[^18^F]FDG since it can be widely used in nuclear medicine units where a cyclotron and an advanced radiochemistry laboratory are not available.

Our study has some limitations. First, it lacks multicentre external validation, not allowing an out-of-sample estimation of the performance of the classifiers, although the use of a test set including 10% of ALS patients represents a valid test set strategy. Second, the transferability of the methodology to other centres may find some obstacles related to the differences in camera type and acquisition protocols. However, to facilitate this transfer the clusters and the statistical methods will be made available to all interested researchers. Third, we did not perform partial volume effect correction for cortical atrophy. Unfortunately, brain MRI scans were not available for all subjects. However, studies employing voxel-based atrophy correction of resting glucose metabolism showed that metabolic measurements were relatively independent of brain atrophy [28]

The main strength of our work is that the explainability-oriented pattern recognition pipeline shows that it is possible not only to predict the survival time at individual patient level from a single brain PET scan but also to identify brain regions, i.e., clusters of voxels, which have been validated a posteriori, that are characteristic of each of the three survival time classes and bear pathophysiological significance. The possibility of opening the “black-box” of learning machines is indeed of paramount importance for physicians to understand the rationale behind the prediction of a pattern recognition algorithm, ensuring transparency and trustworthiness [29].

## Conclusion

We developed a data-driven model based on a single biomarker, which is able to predict the survival range of ALS patients with high accuracy, particularly for the long-surviving class. The lack of a multicentre external validation of the model warrants further studies to evaluate its generalization capability. Within the framework of precision medicine, the advantages of the implementation of this tool are three-fold: first, supporting healthcare professionals in patient management and follow-up; second, facilitating patients’ and caregivers’ life planning, provided their will to be informed is respected; third, improving the design of clinical trials through patients’ stratification according to predicted prognosis.

## Supplementary information

ESM 1 (PDF 402 kb)

## Data Availability

The predictive algorithm and the NIfTI files of the discriminant clusters will be available on demand by interested researchers.
