# Supplementary material for: Role of brain 2-[18F]fluoro-2-deoxy-D-glucose-positron-emission tomography as survival predictor in amyotrophic lateral sclerosis
Source: Eur J Nucl Med Mol Imaging. 2022 Oct 29;50(3):784–91. doi: 10.1007/s00259-022-05987-3 (PMC9852209; doi:10.1007/s00259-022-05987-3)
Supplement: Supplementary file 1 — (PDF 402 kb) [file 259_2022_5987_MOESM1_ESM.pdf]

Article title

Role of brain 2-[^{18}F]fluoro-2-deoxy-D-glucose-Positron-Emission Tomography as survival predictor in Amyotrophic Lateral Sclerosis

Authors

Antonio Canosa, MD, PhD;* Alessio Martino, PhD;* Umberto Manera, MD; Rosario Vasta, MD; Maurizio Grassano, MD; Francesca Palumbo, MD; Sara Cabras, MD; Francesca Di Pede, MD; Vincenzo Arena, MD; Cristina Moglia, MD, PhD; Alessandro Giuliani, PhD; Andrea Calvo, MD, PhD; Adriano Chiò, MD, FAAN;[§] Marco Pagani, MD, PhD[§]

Affiliations

ALS Centre, 'Rita Levi Montalcini' Department of Neuroscience, University of Turin, Turin, Italy (Antonio Canosa, Umberto Manera, Rosario Vasta, Maurizio Grassano, Francesca Palumbo, Sara Cabras, Francesca Di Pede, Cristina Moglia, Andrea Calvo, Adriano Chiò).

Azienda Ospedaliero-Universitaria Città della Salute e della Scienza di Torino, SC Neurologia 1U, Turin, Italy (Antonio Canosa, Umberto Manera, Cristina Moglia, Andrea Calvo, Adriano Chiò).

Institute of Cognitive Sciences and Technologies, C.N.R., Rome, Italy (Antonio Canosa, Alessio Martino, Adriano Chiò, Marco Pagani).

Department of Business and Management, LUISS University, Rome, Italy (Alessio Martino).

Positron Emission Tomography Centre AFFIDEA-IRMET S.p.A., Turin, Italy (Vincenzo Arena).

Environment and Health Department, Istituto Superiore di Sanità, Rome, Italy (Alessandro Giuliani).

Neuroscience Institute of Turin (NIT), Turin, Italy (Andrea Calvo, Adriano Chiò).

Department of Medical Radiation Physics and Nuclear Medicine, Karolinska University Hospital, Stockholm, Sweden (Marco Pagani).

* These authors equally contributed

§ These authors equally contributed

Corresponding author

Antonio Canosa, MD, PhD

ALS Centre, ‘Rita Levi Montalcini’ Department of Neuroscience, University of Turin

Via Cherasco 15, Turin, Italy, 10126

Phone +39116335439

Fax +39116336454

antonio.canosa@unito.it

ORCID ID: https://orcid.org/0000-0001-5876-4079

Supplementary Materials

1. **Notes on ANOVA**
2. **Mathematical derivation of Variance Explained**
3. **On the choice behind Laplacian scores**
4. **Classifiers comparisons, eTable 1**
5. **On the choice behind Informedness**
6. **Supplementary figures (eFigure 1, eFigure 2, eFigure 3)**
7. **References**

1. Notes on ANOVA

The one-way ANOVA (1) is used to determine whether there are any statistical significant differences between the mean survival times of two or more independent (unrelated) groups. As anticipated in the main manuscript, two different ANOVA tests were performed (both of which see the survival time as a continuous response variable):

- Grouping by sex: male or female
- Grouping by onset: spinal or bulbar

The ANOVA test returns the F-statistic, which is the ratio of the between-groups and within-groups variances, and the p-value, which is the probability that the F-statistics can take a value larger than the computed test-statistic value.

2. Mathematical derivation of Variance Explained

Let:

$$C_{i,j} = \left(\sqrt{x_i - \mu_j}\ \right)^2$$

be a matrix encoding the pairwise squared Euclidean distance between the ith sample x_i (i = 1, . . . , n with n being the number of samples) and the mean of the jth group μ_j (j = 1, . . . , k with k being the number of groups – or bins). Further, let:

$$m_i = \min_j C_{i,j}$$

be a vector containing the minimum sample-to-mean distance. The total within- cluster sum-of-squares (TWCSS) reads as:

$$TWCSS = \sum_{i=1}^{n} \quad m_i$$

and the total sum-of-squares (TSS) reads as:

$$TSS = \frac{1}{n}\sum_{i=1}^{n} \ \sum_{j=1}^{n} \ \left(\sqrt{x_i - \mu_j}\ \right)^2$$

Finally, the variance explained (VE), expressed in percentage, reads as:

$$VE = 100\ \cdot \frac{TSS - TWCSS}{TSS}$$

3. On the choice behind Laplacian scores

In the technical literature there exist several strategies for performing feature selection, mainly divided into *filter methods* and *wrapper methods*.

In the former case, features are ranked according to a certain criterion (2-7) and only top-ranked features are retained, whereas irrelevant features are filtered out, hence the name. Conversely, wrapper methods perform a search across combinations to find the best subsets

amongst all features. Since a complete search is often time-consuming, heuristic optimization techniques are frequently utilized to explore candidate solutions (8-12). In our case, since the search space is huge (226954 candidate features), evolutionary or greedy heuristics are likely to fail to converge to a suitable solution due to high probability of being trapped in local solutions. To this end, our choice fell on leveraging filter methods and, in particular, on Laplacian Score (3).

4. Classifiers comparison

In the main manuscript it has been anticipated that Support Vector Machines emerged as the most performing classification system from a comparison amongst Support Vector Machines, K-Nearest Neighbours, Linear Classifier, Decision Tree and Random Forest. We show (eTable 1) the error rate evaluated on the test set for each classifier on each of the three survival time classes. It is worth remarking that all classifiers underwent the Bayesian optimization phase for hyperparameters tuning as described in the main manuscript. Optimization and classification have been fully performed in MATLAB using the Statistics and Machine Learning Toolbox.

eTable 1
Error rate on the test set (in percentage) on the subset of clusters with at least 100 voxels.

Classifier	Class 1	Class 2	Class 3
**Support Vector Machines**	19.51	24.39	4.87
**K-Nearest Neighbours**	21.95	19.51	7.31
**Linear Classifier**	39.02	29.27	9.76
**Decision Tree**	29.26	21.95	12.19
**Random Forest**	26.82	31.70	26.82

5. On the choice behind Informedness

The informedness J, also known as Youden's Index (13), is a robust statistic that summarizes the outcome of a diagnostic test and is defined as:

$$J = Specificity + Sensitivity - 1$$

where, in turn:

$$Sensitivity = \frac{true\,positives}{true\,positives + false\,negatives}$$

$$Specificity = \frac{true\,negatives}{true\,negatives + false\,positives}$$

By definition $J \in [-1, +1]$ and, being positive-oriented, the higher, the better. In fact, when $J = 1$, the test is perfect (i.e., there are no false positives and no false negatives). The informedness represents the probability of an informed decision by the prediction algorithm, as opposed by a random guess (which would yield $J = 0$). The choice behind the informedness over other performance indices stems from the fact that the informedness is, along with the Matthews Correlation Coefficient, the most robust and informative performance index, especially in cases of unbalanced data (14). In fact, many common performance measures (e.g., accuracy, F-measure, and the like) either do not take into account all predictions (whereas the informedness considers all of them) or show a bias towards the majority class when the dataset is unbalanced.

6. Supplementary figures

eFigure 1

Kaplan-Meier estimate of survival functions.

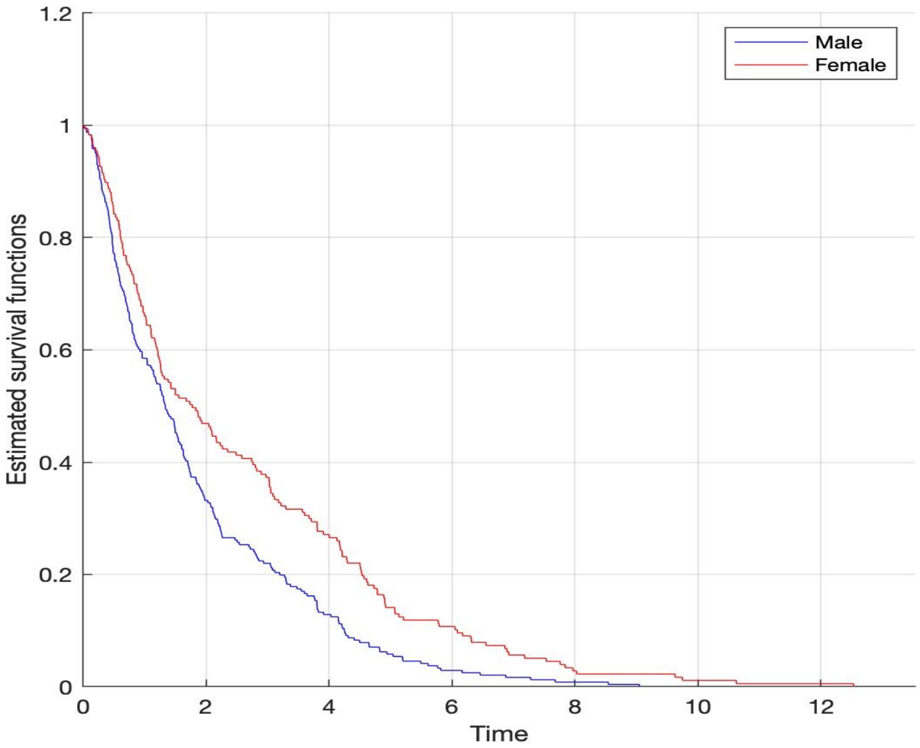

eFigure 2

Survival Time distribution within the dataset

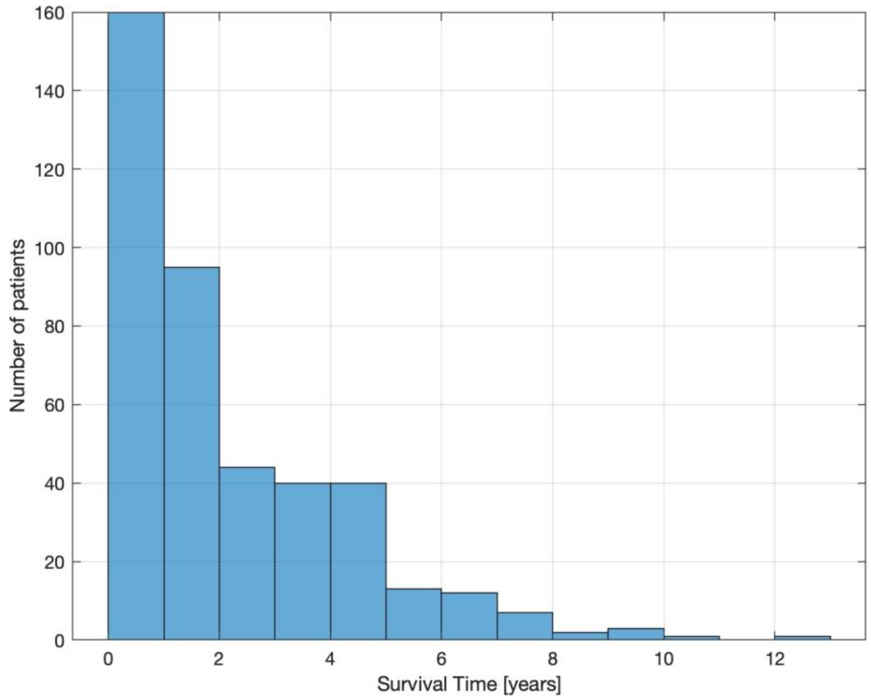

eFigure 3

Box plot of the observations for each of the four King's stages: *F*=4.18, *p*=0.0062

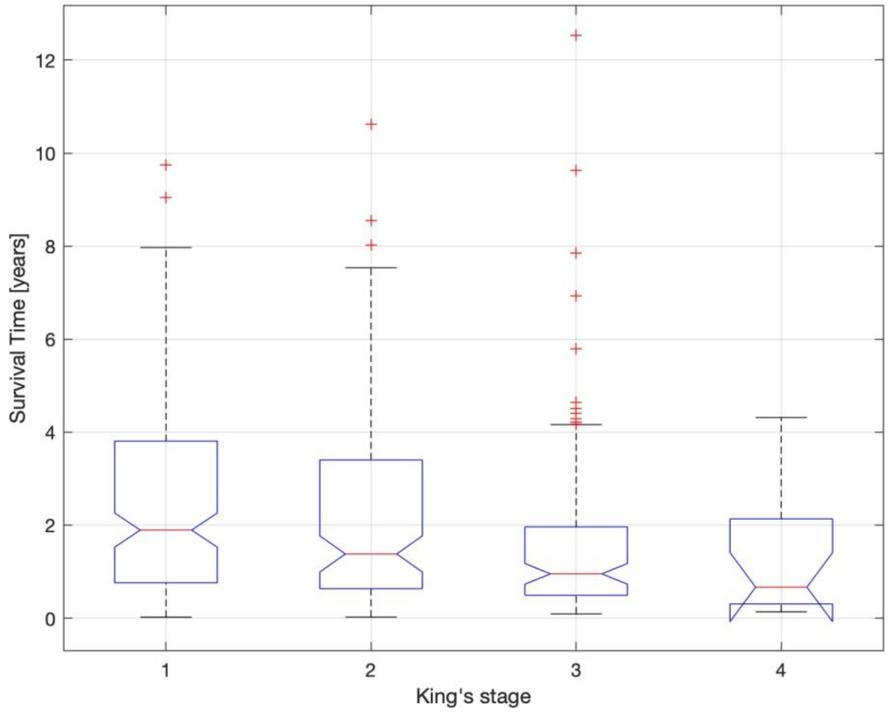

7. References

1. Hogg RV, & Ledolter J. (1987). *Engineering statistics*. Macmillan Publishing Company.

2. Dash M, Choi K, Scheuermann P, Liu H. (2002). Feature selection for clustering-a filter solution. In *2002 IEEE International Conference on Data Mining, 2002. Proceedings.* (pp. 115-122). IEEE.

3. He X, Cai D, Niyogi P. (2005). Laplacian score for feature selection. In *Proceedings of the 18th International Conference on Neural Information Processing Systems* (pp. 507-514).

4. Talavera L. (1999). Feature Selection as a Preprocessing Step for Hierarchical Clustering. In *Proceedings of the Sixteenth International Conference on Machine Learning* (pp. 389-397).

5. Theodoridis S, Koutroumbas K. (2008) *Pattern recognition*. Academic Press. 4th edition.

6. Zhao Z, Liu H. (2007). Spectral feature selection for supervised and unsupervised learning. In *Proceedings of the 24th international conference on Machine learning* (pp. 1151-1157).

7. Zhao ZA, Liu H. (2012). *Spectral Feature Selection for Data Mining*. Taylor & Francis. 1st edition.

8. Zhu W, Wang X, Ma Y, Rao M, Glimm J, Kovach JS. (2003). Detection of cancer-specific markers amid massive mass spectral data. *Proceedings of the National Academy of Sciences*, *100*(25), 14666-14671.

9. Dy JG, Brodley CE. (2004). Feature selection for unsupervised learning. *Journal of machine learning research*, *5*(Aug), 845-889.

10. Kim Y, Street WN, Menczer F. (2002). Evolutionary model selection in unsupervised learning. *Intelligent data analysis*, *6*(6), 531-556.

11. Martino A, Giuliani A, Rizzi A. (2019). (Hyper) graph embedding and classification via simplicial complexes. *Algorithms*, *12*(11), 223.

12. Martino A, Giuliani A, Todde V, Bizzarri M, Rizzi A. (2020). Metabolic networks classification and knowledge discovery by information granulation. *Computational biology and chemistry, 84*, 107187.

13. Youden WJ. (1950). Index for rating diagnostic tests. *Cancer*, *3*(1), 32-35.

14. Powers DMW. (2011). Evaluation: From Precision, Recall and F-measure to ROC, Informedness, Markedness & Correlation. *J. Mach. Learn. Technol*, *2*(1), 37-63.
